# Identification and construction of a multi-epitopes vaccine design against *Klebsiella aerogenes*: molecular modeling study

**DOI:** 10.1038/s41598-022-18610-0

**Published:** 2022-08-24

**Authors:** Sami I. Alzarea

**Affiliations:** Department of Pharmacology, College of Pharmacy, Jouf University, Sakaka, Aljouf 72341 Saudi Arabia

**Keywords:** Peptide vaccines, Protein design, Bacteria

## Abstract

A rapid rise in antibiotic resistance by bacterial pathogens is due to these pathogens adaptation to the changing environmental conditions. Antibiotic resistance infections can be reduced by a number of ways such as development of safe and effective vaccine. *Klebsiella aerogene* is a gram-negative, rod-shaped bacterium resistant to a variety of antibiotics and no commercial vaccine is available against the pathogen. Identifying antigens that can be easily evaluated experimentally would be crucial to successfully vaccine development. Reverse vaccinology (RV) was used to identify vaccine candidates based on complete pathogen proteomic information. The fully sequenced proteomes include 44,115 total proteins of which 43,316 are redundant and 799 are non-redundant. Subcellular localization showed that only 1 protein in extracellular matrix, 7 were found in outer-membrane proteins, and 27 in the periplasm space. A total of 3 proteins were found virulent. Next in the B-cell-derived T-cell epitopes mapping phase, the 3 proteins (Fe2^+−^ enterobactin, ABC transporter substrate-binding protein, and fimbriae biogenesis outer membrane usher protein) were tested positive for antigenicity, toxicity, and solubility. GPGPG linkers were used to prepare a vaccine construct composed of 7 epitopes and an adjuvant of toxin B subunit (CTBS). Molecular docking of vaccine construct with major histocompatibility-I (MHC-I), major histocompatibility-II (MHC-II), and Toll-like receptor 4 (TLR4) revealed vaccine robust interactions and stable binding pose to the receptors. By using molecular dynamics simulations, the vaccine-receptors complexes unveiled stable dynamics and uniform root mean square deviation (rmsd). Further, binding energies of complex were computed that again depicted strong intermolecular bindings and formation of stable conformation.

## Introduction

Infections caused by bacteria are treated with antibiotics. As a defense mechanism, antibiotic resistance occurs in microorganisms like bacteria, fungi, or viruses over time to adapt the environment and protect themselves against antibiotics therapy^1^. As a result of the overuse and misuse of antibiotics, this phenomenon has been accelerated. It is a serious global health threat that causes human and animal mortality and morbidity at alarming rates^2^. By adapting to changing environmental conditions, antibiotic resistance is the outcome of bacterial evolution. In this regard, new approaches to manage antibiotic resistance pathogens are urgently needed^3^. Biological preparations can be utilized to boost immunity of host to any infection^4^. A good vaccine supply is the only way left to better control antibiotic resistance bacterial pathogens. Potential vaccine candidates must fulfill specific requirements in order to develop a successful vaccine, such as being highly antigenic, conserved, and non-homologous to the host normal flora or host. By applying Pasteur's vaccinology concept, Salk and Sabin developed a safe and effective vaccine against poliovirus. A few years later, Pasteur developed the BCG vaccine against tuberculosis, together with vaccines against measles, mumps, and rubella^5^. Such vaccination is, however, rendered useless in the case of pathogens that cannot be grown or cultured in the laboratory^6^. The antigenic variability of culture-based vaccines was also notable. *Neisseria meningitides* and *Mycobacterium leprae* have posed particular challenges during vaccine development using Pasteur vaccinology. Essential parameters of vaccines must be met to make an effective vaccine^7^. An up to date reverse vaccinology is based genomic information to prioritize potential vaccine targets without the need for culture^8^. Antibodies specific to pathogens can be designed and developed via immunotherapy; at the same time, the human immune system can be trained to acquire adaptive immunity in the fight against bacterial infections. In particular, the last strategy uses vaccines to reduce the number of antibiotic resistance infections^9^. The traditional vaccinology costs a lot of money, takes a lot of time, and needs a large number of human resources^10^. Using genome based reverse vaccinology technique is quite beneficial to unveil new vaccine targets that are difficult to discover through traditional vaccine development^11^. Antigens can be identified through vast variety of genomic data generated from next-generation sequencing (NGS) data. The reverse vaccinology approach was successfully used to develop the meningococcal serogroup B vaccine (4CMenB)^12^.

*Klebsiella aerogenes* is a Gram-negative, motile and rod-shaped bacterium that belongs to the family of Enterobacteriaceae. It was formerly known as *Enterobacter aerogenes* according to Hormaeche and Edwards^13^. Nosocomial outbreaks are frequently associated with *K aerogenes*. A variety of infections associated with the pathogen include tissues and skin infections, urinary, bloodstream and respiratory infections^14^. There is widespread knowledge about it being involved surgical infections, bacteremia, pneumonia, meningitis, endocarditis, infections of the eye, intra-abdominal infections, infections of the central nervous system, septic arthritis and osteomyelitis. A higher death rate in ICU patients is associated with strains of *K. aerogenes* which are resistant to multiple drugs. This specie is responsible for a high mortality rate and morbidity rate in the recent past^15^.

*K. aerogenes* infection rates in hospitals have increased due to the overuse of antibiotics^16^. A wide variety of antibiotics are known to be resistant to said bacteria. A trend of increasing *K. aerogenes* isolates has been observed^17^. The bacteria *K. aerogenes* is resistant to beta-lactams and antibiotics that are broad-spectrum, as previously reported^18^.We identified promising vaccine targets by employing a systematic *"*in silico*"* approach. Computing methods and omics data have proved to be advantageous in recent years when it comes to finding new vaccine targets and reducing vaccine failure rates in clinical trials^19^.

## Methodology

**Figure 1:**
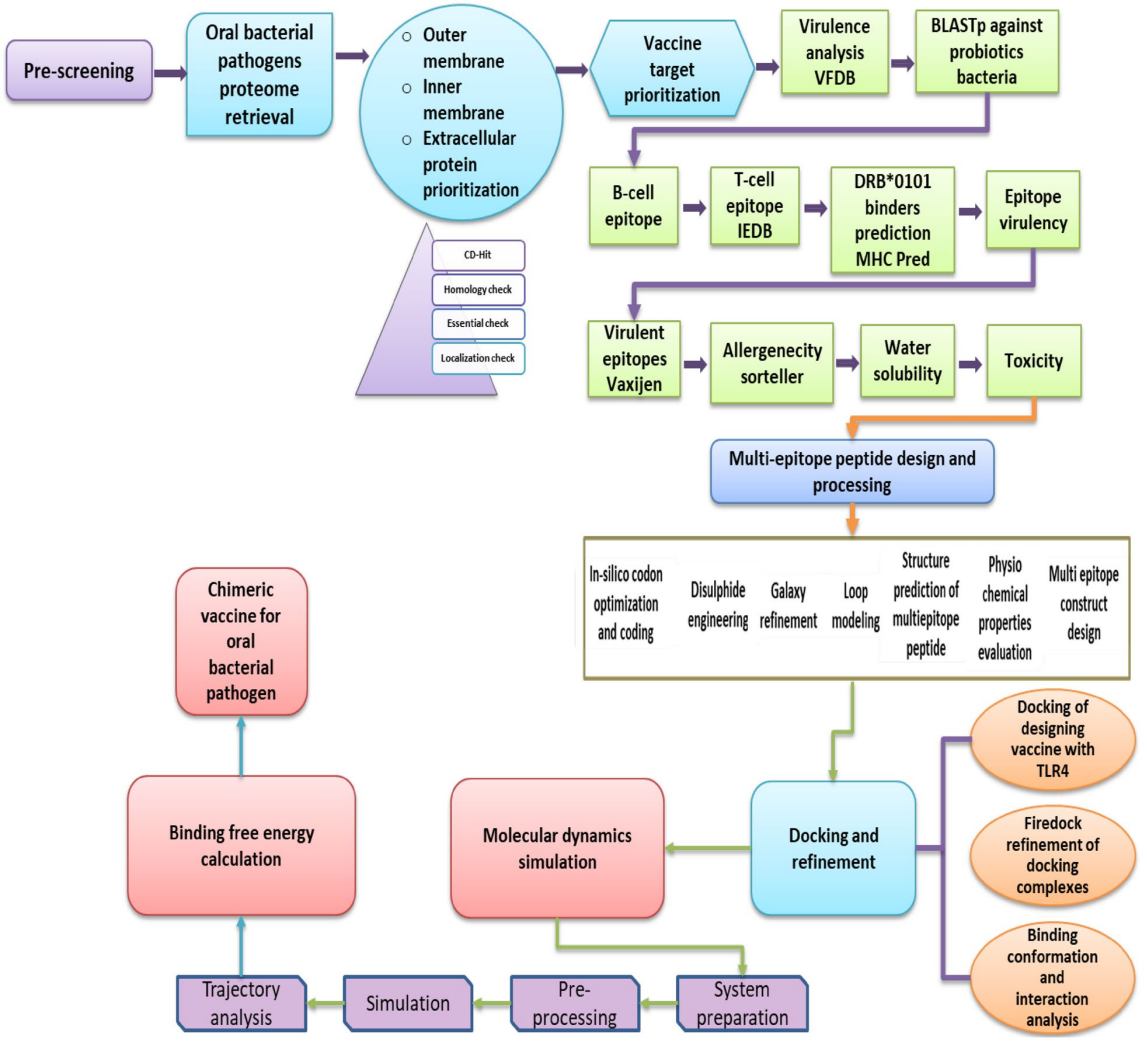
A complete work flow of the study.

A methodology flow is illustrated in Fig. 1 with illustrates the steps used for development of a multi-epitopes vaccine against *K. aerogenes*.

### Complete proteome retrieval

Proteomes of *K. aerogenes* were initially retrieved in order to begin this study. Proteomic information of seventeen strains of the bacteria were analyzed from the National Center for Biotechnology Information's genome database (NCBI)^20^. FASTA files were retrieved and subjected to further analysis^21^.

### Bacterial pan-genome analysis (BPGA) tool analysis

BPGA is an important bioinformatics tool for the identification of core, unique, and accessory proteins. This study investigated the potential pan-genome of the pathogen by analyzing all available fully sequenced genomes^22^. All 17 strains of *K. aerogenes* were investigated by the BPGA tool^43^ using one-click analysis to identify core genome (conserved), as well as strain-specific proteins. The cut-off similarity percentage allowed was 50%.

### Pre-screening phase

Pre-screening is the filtering/subtractive proteomic phase for prioritization of vaccine candidates during which possible antigenic targets were recognized in the core genome of the pathogen. As part of this phase, the analysis of CD-Hit^23^, the non-homology validation between pathogen and host proteome, the essential analysis, and the identification of surface protein was done.

### Cluster data with high identity and tolerance (CD-Hit) analysis

Among the protein sequences in the bacterial core genome, there are a lot of redundant and non-redundant sequences. Double representation of redundant proteins in the proteome's is of no importance in vaccine designing. An analysis using the CD-Hit-h algorithm was used to eliminate all redundant proteins. A second processing method was considered for non-redundant proteins^24^.

### Subcellular localization analysis

Surface-localized proteins were prioritized as vaccine candidates due to their capability of being easily recognized by the host immune system. PSORTb 3.0 analysis provided the solution for such tasks^25^. Only extracellular, outer membrane and periplasmic proteins were selected while other were not processed.

### Virulent protein analysis

A dataset of virulence factors for bacteria is the virulent factor database (VFDB). We analyze the prioritized subcellular localized proteins for virulence using Basic Local Alignment Search (BLASTp) against VFDB complete proteome dataset^26^. The Cut-off values remained 100 for bit score and 30% of sequence identity. A protein that did not meet the mentioned criteria was dropped out^23^.

### Analysis of BLASTp against microbiome and humans

The proteins homology was compared using BLASTp. The filtered virulent proteins were compared to the human proteome and normal flora. To prevent autoimmune responses to self-antigens, a homology check was performed. The selected proteins were BLASTp against humans and three different types of bacteria: *Lactobacillus casei* (taxid number: 1582), *L. johnsonii* (taxid number: 33959) and *L. rhamnosus* (taxid number: 47715)^27^. A cut-off E-value of 10^−4^ and sequence identify of ≤ 30% were used.

### Vaccine epitopes prioritization phase

The proteins were investigated further for B-cell and T-cell epitopes prediction. The epitopes then underwent physicochemical analysis, the number of transmembrane helices analysis, antigenicity, allergenicity and adhesion probability prediction^28^.

### Physiochemical analysis

The physiochemical characteristics of the selected proteins were analyzed, including instability index, molecular weight, atomic composition, theoretical protein index, aliphatic index, amino acid composition, and the grand average of hydropathicity (GRAVY).ProtParam^29^ was used to calculate the physicochemical properties of the proteins. Protein molecular weight was cut-off at 110 KDa and the instability index was < 40^30^. This was used to identify the protein as an appropriate vaccine target. Stable proteins were analyzed further, while unstable proteins were removed from the study.

### Transmembrane helices

A transmembrane helices detection was done with HMMTOP 2.0 and TMHMM 2.0^31^. Numbers less than 0 or 1 of transmembrane helices were selected. Only proteins having transmembrane helices less than the threshold were used as such proteins can easily cloned and purify^32^.

### Antigenicity and allergenicity prediction

An antigenicity refers to proteins ability to attach with immune cells and produces the appropriate immune responses. Through VaxiJen 2.0, a cut-off value of ≥ 0.4 was used to determine potential antigen vaccine candidates. An antigenic vaccine may provoke the host immune system more effectively^33^. These potential candidate must pass through allergenicity check through online server named allertop 2.0^34^.

### Adhesion probability analysis

The following step was to just select and prioritize proteins with an ability to adhere based on their adhesion probability. It was determined that adhesive candidates should have a value over 0.5. A robust host immunity can be ensured when the vaccine is actively attached to the host immune cells. For an adaptive immunity response, antibodies and TCR (T-cell receptors) are produced through adhesion and interaction with the immune system receptors of the host. Web server from Vaxigen was used to accomplish this task^35^.

### Epitopes prediction phase

To anticipate B-cell and T-cell epitopes, the immune-epitope database (IEDB) was employed^36^. Immune epitopes of all types are featured in the IEDB and they can be easily accessed. The first step was to predict B-cell epitopes and then to use these epitopes to predict T-cell epitopes. A low percentile rank was used to prioritize predicted epitopes. A binding potency analysis was also performed through the MHC-Pred tool for DRB*10,101 allele-containing putative epitopes^37^. VaxiJen 2.0 was used to examine the antigenicity of selected epitopes i.e., Virulent-Pred, ToxinPred, and proteins to determine their antigenicity, toxicity, virulence, and water solubility, respectively^27^.

### Multi-epitope vaccine designing and processing

Multiple antigenic epitopes can be combined in multi-epitope vaccines. Following the screening of all epitopes and the usage of GPGPG linkers^38^, the multi-epitope vaccine was created. Cholera toxin B subunit (adjuvant)^39^ was added to the vaccine as adjuvant to boost immunogenicity.

To determine the most stable vaccine structure that will allow molecular recognition, we modeled the vaccine construct using the 3Dpro Scratch tool. The design's stability and durability were assured by inspecting its 3D structure^40^.

### Loops modeling and galaxy refinement

A Galaxy loop tool of the Galaxy web server was used to model the vaccine construct to remove unnecessary loops^41^. Galaxy Refine v 2.0 was then used to analyze the loop-modeled vaccine construct and steric clashes were removed from the vaccine and its side chains were reconstructed. Refined vaccine construct was deemed good and promising vaccine candidate^42,43^.

### Disulfide engineering

To improve the vaccine's stability, a disulfide engineering approach was used. Reduced conformational energy increased the stability of the vaccine. The outer and inner chain bonds were treated to enhance and improve stability. The Design 2.0 webserver was used to undertake disulfide engineering^44,45^.

### Codon optimization

The multi-epitope vaccine's DNA sequence was generated and transformed to *Escherichia coli* expression system utilizing the Java Codon Adaptation Tool (JCat) platform. The vaccine expression in *E. coli* was measured through the codon adaptation index (CAI) and the vaccine's GC percentage^46^.

### Molecular docking

The vaccine construct was docked with a variety of immune cell receptors in order to determine how effective it will be in generating an immune response^47^. The vaccine's binding affinity was predicted in a blind docking study for TLR-4 (4G8A), MHC-I (1I1Y), and MHC-II (1KG0) receptors^46^. Using PatchDock^48^ docking investigation was accomplished followed by FireDock server to refine the docked complexes^49^.

### Molecular dynamics simulation

Molecular dynamics simulations were performed using AMBER20 software. Antechamber was employed for preprocessing of the complexes and TIP3P water boxes were used for submersion of vaccine–receptor complexes. FF14SB force field was served as force field for docked complexes. Hydrogen bonds were constrained by the SHAKE algorithm^50^. An equilibration was conducted for system for 1 ns and followed by a production run were performed for 100 ns. The trajectories were examined using CPPTRAJ module. In addition, the AMBER MMPBSA.py module was utilized to assess the vaccine's and receptor's intermolecular affinity^51^ by examining only 100 frames.

### C-Immune simulation

For deciphering host-immune responses to the designed vaccine, the C-ImmSim server was used^52^. Antigens on this server were characterized and their profiles calculated to determine the immune responses of the host, which in this case is the human body.

## Results

### Complete proteome retrieval

As part of the present research, the NCBI databases (https://www.ncbi.nlm.nih.gov/) were consulted to retrieve the complete proteome of 17 strains of *K. aerogenes* followed by a complete pan-genome analysis. GC content of these pathogens varies from 54.46 to 57.06, while strain size ranges from 5.09 Mb to 5.81 Mb. Table 1 describes strain's genome size, type, and GC content of the strains.

**Table 1:** Genomics statistics of *K. aerogenes* strains.

S.no	Organism name	Strain	Size (Mb)	GC%
1	*K. aerogenes*	FDAARGOS 1442	5.28	54.90
2	*K. aerogenes*	NCTC9644	5.81	57.06
3	*K. aerogenes*	RHBSTW-00898	5.45	54.83
4	*K. aerogenes*	AR_0009	5.33	55.00
5	*K. aerogenes*	AR_0007	5.12	55.10
6	*K. aerogenes*	MINF_10B-sc-2280448	5.12	55.00
7	*K. aerogenes*	AR_0018	5.09	55.20
8	*K. aerogenes*	035	5.38	54.46
9	*K. aerogenes*	AR_0062	5.45	54.89
10	*K. aerogenes*	HNHF1	5.49	54.73
11	*K. aerogenes*	FDAARGOS 1441	5.19	55.00
12	*K. aerogenes*	NCTC9735	5.19	55.00
13	*K. aerogenes*	WP5-W18-CRE-01	5.46	54.78
14	*K. aerogenes*	AR_0161	5.54	54.65
15	*K. aerogenes*	NCTC10006	5.27	54.91
16	*K. aerogenes*	EA1509E	5.59	54.93
17	*K. aerogenes*	KCTC 2190	5.28	54.80

### BPGA analysis

**Figure 2:**
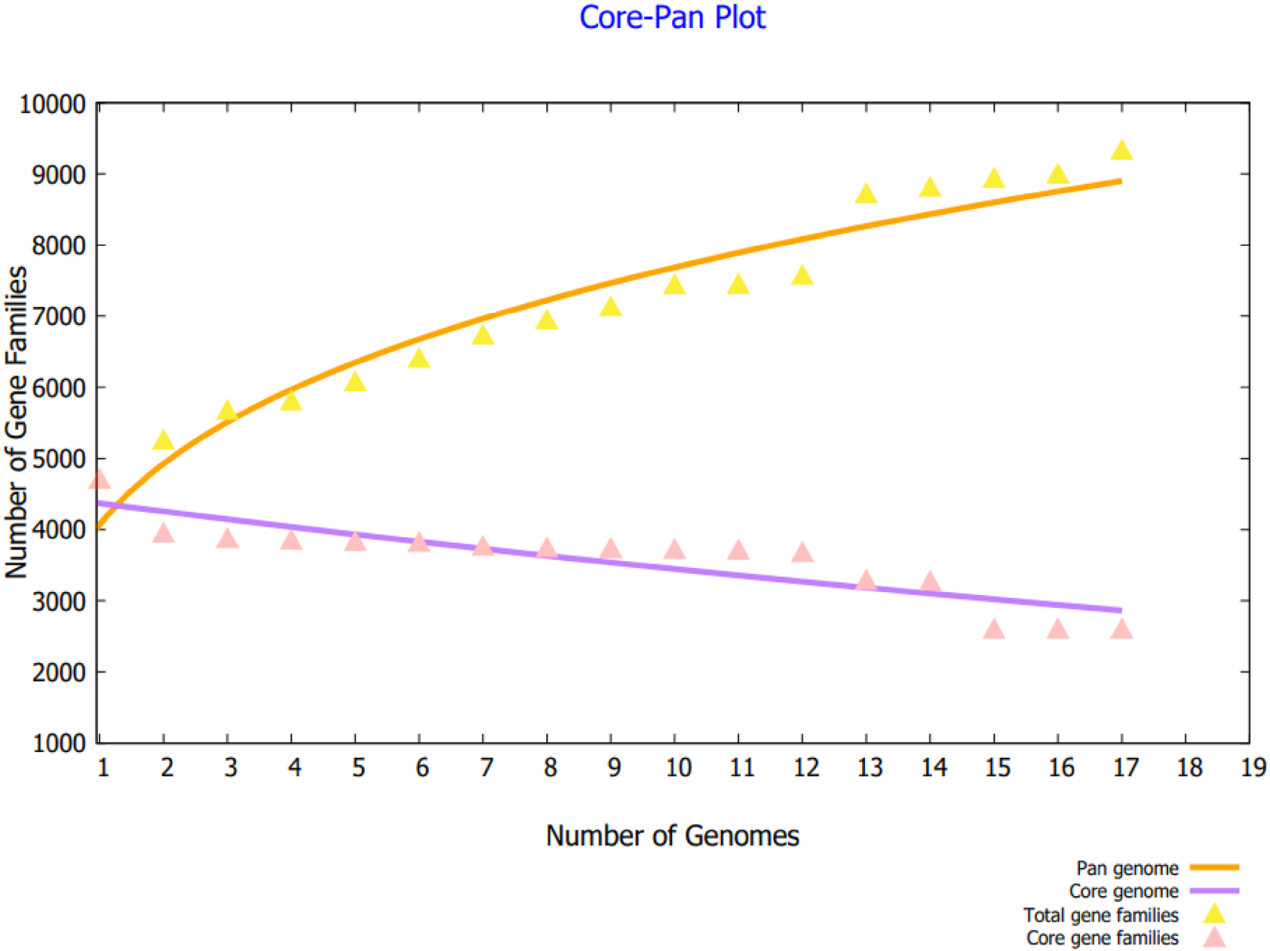
Core pan plot of *K. aerogenes* strains.

**Figure 3:**
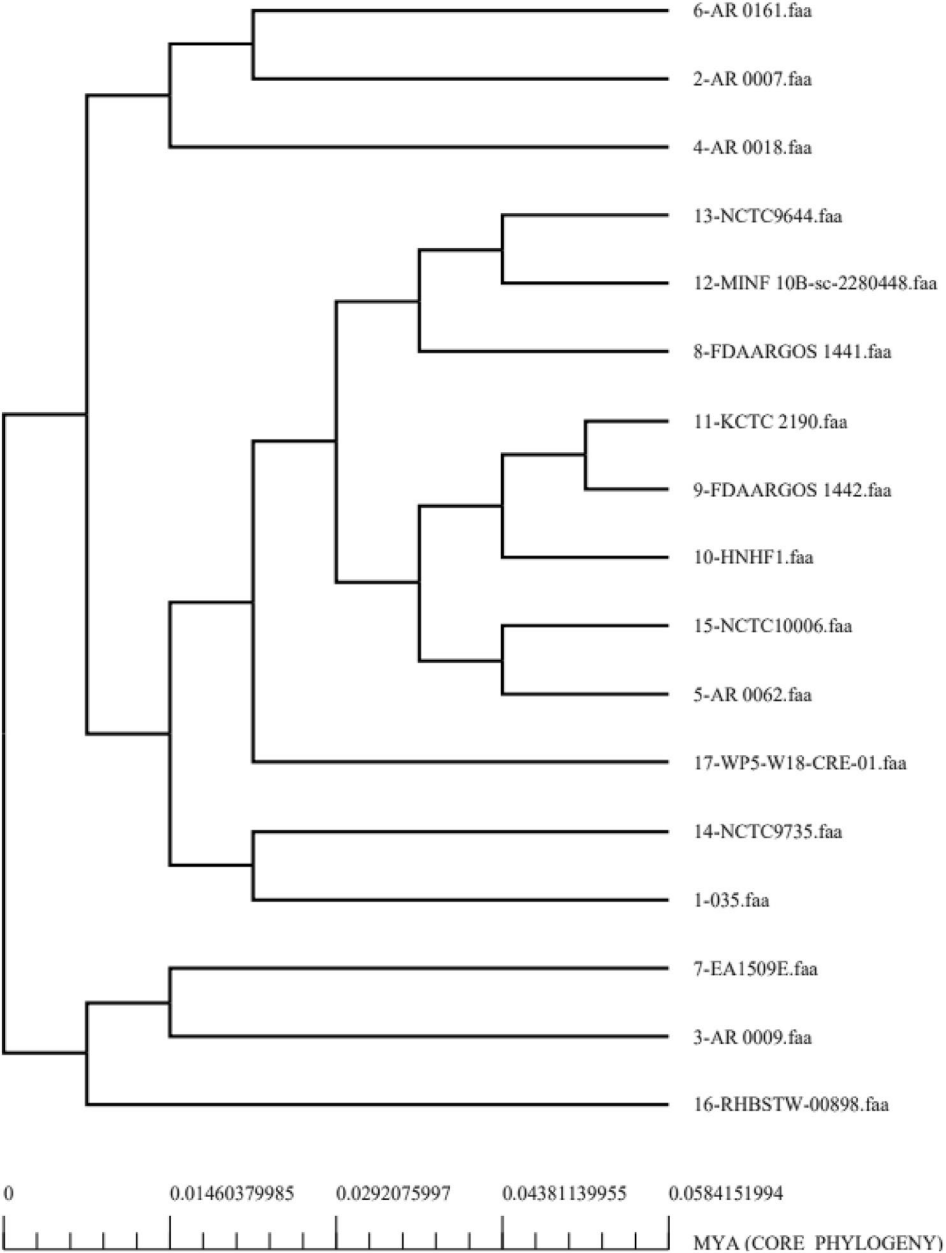
*K. aerogenes* strains phylogenetic tree.

In Fig. 2, each of the 17 strain is presented against the number of gene families. Considering the open nature of pathogen genomes, which can be seen in the core-pan plot, there is a very high probability of gaining new genes over time as a result of genome plasticity. Further, the core proteins also play a role in metabolic regulation and metabolic biogenesis as demonstrated by the distribution analysis of COGs. Information is stored and processed by genes, primarily via unique proteins. As part of the core genome, RNA is processed, and the recombination genes are duplicated, translated, and transcribed. Figure 3 shows the pan-phylogenetic tree for all 17 K*. aerogenes* species.

### CD-HIT Analysis

**Figure 4:**
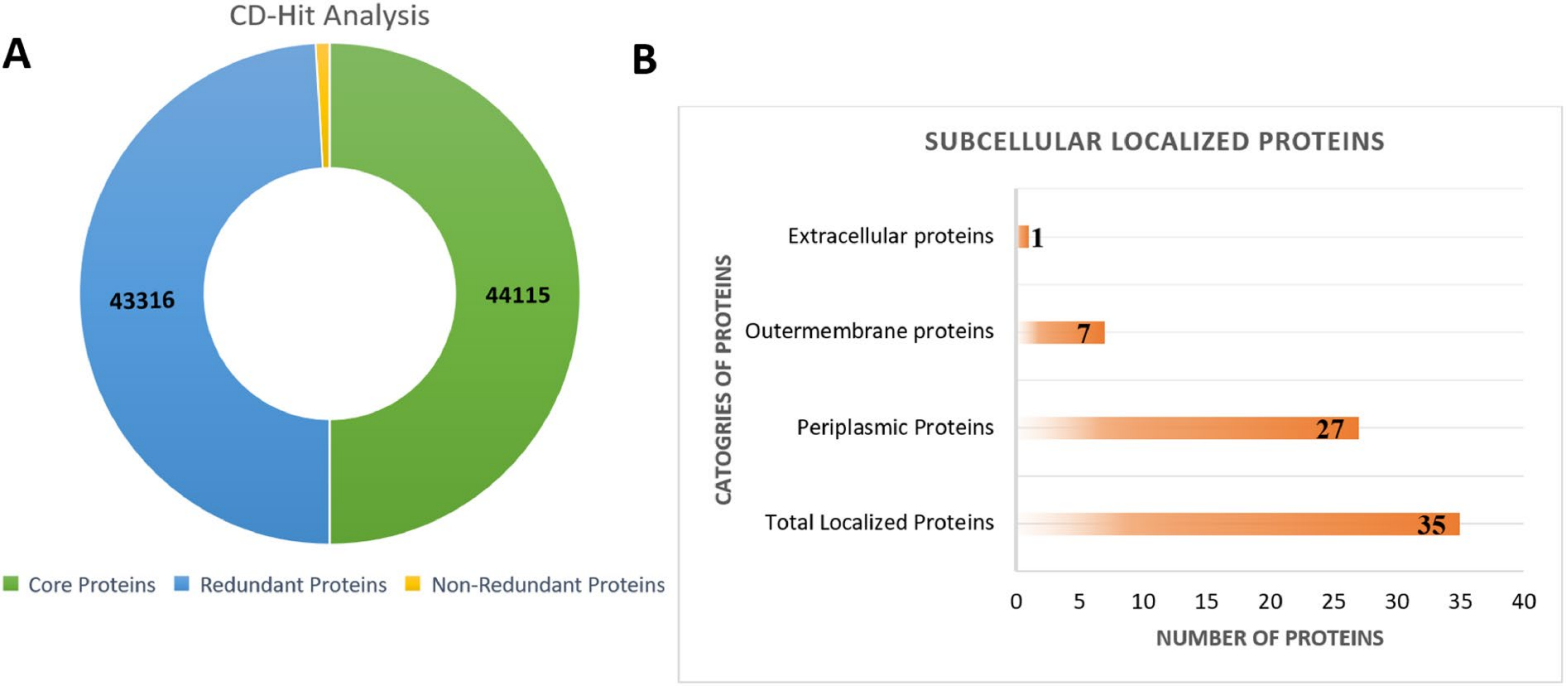
(**A**) Shows the percentage of the total proteome, core proteome, redundant and non-redundant proteins. (**B**) Several proteins can be found in the extracellular, periplasmic, and extracellular membranes.

The CD-hit analysis was employed to retrieve core proteome sequences without duplicates^23^. According to Fig. 4A, 799 of *K. aerogenes* proteome was non-redundant, while 43,316 were redundant while the whole proteome has 44,115 proteins. In this study, redundant protein sequences were eliminated because the repetitions of the same proteins were not necessary for vaccine development, while the redundancy-free core proteins were used in the subcellular localization phase and virulent analysis.

### Subcellular localization

When using surface or membrane proteins in the design of a vaccine, the immune system is able to recognize them easily; therefore, potential immune responses are generated. Surface proteins should also be subjected to subcellular localization analysis^25^. A subcellular localization analysis revealed total 35 proteins in which we have 27 periplasmic membrane proteins in the non-redundant core proteome, extracellular is only one in number while outer membrane proteins are seven in number shown in Fig. 4B.

### VFDB analysis

Infectious pathogens are mainly characterized by the presence of virulent proteins^53^. The VFDB analysis defined these protein sequences as virulent if bit score > 100% and sequence identity is greater than 30%. Three proteins sequence out of 35 subcellular located proteins were identified as virulent which consists of two periplasmic and one outer membrane protein (Table 2).

**Table 2:** Virulent proteins were identified in the set of exposed proteins.

	Bit score	Sequance identity
**Extracellular proteins**		
> core/1216/1/Org1_Gene2191	249	37%
> core/3201/1/Org1_Gene4005	545	86%
**Outer membrane proteins**		
> core/238/1/Org1_Gene4685	1379	81%

### Human and normal flora, adhesion probability, physiochemical property analysis

All the virulent proteins were examined for homology against the gut flora and human genomes in order to prevent the autoimmune responses. In this study, one protein showed homology with humans and with the flora of the normal host and was discarded. A sequence with more than one transmembrane helix was removed from further analysis^31^, while those with no transmembrane helices or one were considered. No sequence discarded in this step. Only two protein sequences were forwarded for further analysis.

### Vaccine epitopes prioritization phase

A phase of epitope prioritization was carried out for prioritized proteins that passed the above-mentioned steps and checks. It was predicted that both B- and T-cell epitopes would stimulate immune responses during the epitope prioritization phase.

### B-cell epitopes prediction

The process in which B-cells transform into plasma cells after stimulation may also be called humoral immunity. A list of two proteins for B-cell epitope prediction was assembled namely: Fe2^+−^ enterobactin ABC transporter substrate-binding protein and fimbrial biogenesis outer membrane usher protein. Here we first anticipate B-Cell epitopes followed by predicting T-Cell epitopes. Here we predict peptides for two selected proteins (Table 3).

**Table 3:** Predicted B-cell epitopes and shortlisted vaccine targets.

B-cell epitopes	Peptides
> core/3201/1/Org1_Gene4005 Fe2^+−^ enterobactin ABC transporter substrate-binding protein	PNNRVADAQGFLRQWGDVAKKRNLARLYIGEPSAE
	GGDSALALYDQL
	FKLATLPGNLHASQSQGKRHDIVQLG
> core/238/1/Org1_Gene4685 fimbrial biogenesis outer membrane usher protein	LEGSRDNQNDQRI
	RQQLSRLYNEAFNDALKIPLT
	LGTVLRSRSEDIGQSSVKTF
	YNNQMRSGGSNTS
	TWNLQSLGPMTAI
	ASSTVFDNSQSA
	LSVQNFVMGNHEVDTRGLPYG
	RVNKLFTRGRGAGAPLA
	HMDRWSESGKKTQPA
	GYDNNAVGETRITLPLTEAVNINLQNMLASDS
	WINQEKTVIGDKLRRSDADNRAIGG
	WINQEKTVIGDKLRRSDADNRAIGG
	NDDRRYNSHYYTAD
	RYNNGDSNANTG
	LGNWFSAGMTHQNGYTMANISARKQFNEGAIRT
	SRAISGDTGDDKTLSG
	KNSIDSYDIVSGRKS
	LSSWAAVQQTGEG

### T-cell epitopes prediction

T-cell epitopes are primarily responsible for triggering a cellular immune responses. This is known as cellular immunity or T-cell-dependent immunity. Following recognition of peptide antigens, T-cell lymphocytes multiply and differentiate into the primary immune response. Using B-cell epitopes generated from T-cell epitopes, we predicted B-cell-derived T-cell epitopes that are able to activate cellular immunity, and so the lowest percentile scores were used to identify MHC-I and MHC-II epitopes (Table 4). The alleles that belong to the MHC-I subset are as follows: HLA-A*01:01, HLA-A*01:01, HLA-A*02:01, HLA-A*02:01, HLA-A*02:03, LA-A*02:03, HLA-A*02:06, HLA-A*02:06, HLA-A*03:01, HLA-A*03:01, HLA-A*11:01, HLA-A*11:01, HLAA*23:01, HLA-A*23:01, HLA-A*24:02, HLA-A*24:02, HLA-A*26:01, HLA-A*26:01, HLAA*30:01, HLA-A*30:01, HLA-A*30:02, HLA-A*30:02, HLA-A*31:01, HLA-A*31:01, HLAA*32:01, HLA-A*32:01, HLA-A*33:01, HLA-A*33:01, HLA-A*68:01, HLA-A*68:01, HLAA*68:02, HLA-A*68:02, HLA-B*07:02, HLA-B*07:02, HLA-B*08:01, HLA-B*08:01, HLA-B*15:01, HLA-B*15:01, HLA-B*35:01, HLA-B*35:01, HLA-B*40:01, HLA-B*40:01, HLA-B*44:02, HLAB*44:02, HLA-B*44:03, HLA-B*44:03, HLA-B*51:01, HLA-B*51:01, HLA-B*53:01, HLA-B*53:01, HLA-B*57:01, HLA-B*57:01, HLA-B*58:01, HLA-B*58:01; and MHC-II alleles are: HLADRB1*01:01, HLA-DRB1*03: *04:01, HLA-DRB101,HLA-DRB1*04:05, HLA-DRB1*07:01, HLADQA1*03:01/DQB1*03:02, HLADQA1*03:01/DQB1*03:02, HLADQA1*01:02/DQB1*06:02, HLADPA1*02:01/DPB1*01:01, HLADPA1*01:03/DPB1*04:01, HLADPA1*03:01/DPB1*04:02, HLADPA1*02:01/DPB1*05:01, HLADPA1*02:01/DPB1*14:01.

**Table 4:** MHC-I and MHC-II predicted epitopes.

MHC-I	Percentile score	MHC-II	Percentile score
WGDVAKKRNL	6.4	WGDVAKKRNLARLYI	13
DSALALYDQL	3.2	GDSALALYDQL	6.3
KLATLPGNL	0.22	FKLATLPGNLHA	1.6
HASQSQGKR	0.33	LHASQSQGKRHDIVQL	23
QGKRHDIVQL	1.6	RQQLSRLYNEAFND	
RQQLSRLYN	4.4		7.1
RLYNEAFND	12		
LYNEAFNDAL	1.1	LYNEAFNDALKIPLT	7.7
TVLRSRSEDI	16	LGTVLRSRSEDI	2.3
SRSEDIGQSS	5.9	SRSEDIGQSSVKTF	26
TWNLQSLGPM	18	TWNLQSLGPMTAI	1.9
STVFDNSQSA	4.5	SSTVFDNSQSA	9.4
RGRGAGAPLA	12	TRGRGAGAPLA	1.1
AVNINLQNML	15	AVNINLQNMLASDS	0.99
WINQEKTVI	4.9	WINQEKTVIGDKLRRS	7.7
TVIGDKLRRS	2.6		
DDRRYNSHYY	2.2	DDRRYNSHYYTAD	27
NGDSNANTG	30	YNNGDSNANTG	21
YTMANISARK	3.4	NGYTMANISARKQF	1.5
NISARKQFN	42	NISARKQFNEGAIRT	30
IDSYDIVSGR	0.98	IDSYDIVSGRKS	6.5
SSWAAVQQTG	4	LSSWAAVQQTG	2.1

### Epitope prioritization phase

A number of analyses were performed after the epitope prediction in the prioritization phase, including binding affinity assessments using DRB*0101, followed by allergenicity and solubility analysis. In order for the immune system to function properly, vaccines must bind to immune cell receptors. DRB*0101 analysis later investigated the potential to bind with HLA DRB*0101 allele at all selected epitopes^54^. However, only those epitopes that had an IC50 lower than 100 nM were considered for further analysis.

### Antigenicity, allergenicity, solubility, and toxicity analysis of selected epitopes

A host's immune system can only be stimulated by antigenic proteins^33^. All possible non-antigenic sequences of proteins were excluded from the study in order to achieve this aim. For removal of all toxic and allergic proteins and poor water-soluble epitopes, allergenicity and toxicity analyses were performed to avoid allergic and toxic responses^34,41^. InvivoGen was used as a webserver to calculate solubility, which can be accessed at https://www.invivogen.com/ova-peptide. All shortlisted epitopes for multi-epitopes are given in Table 5.

**Table 5:** List of all shortlisted possible antigenic, non-allergenic, nontoxic, and water-soluble peptides.

MHC- Pred	MHC- Pred IC50 value (nM)	Antigenicity	Allergenicity	Solubility	Toxin Pred
GDVAKKRNL	17.1	Antigenic	Non-allergen	Good water solubility	Non-toxic
TVIGDKLRR	14.52				
VIGDKLRRS	22.86				
KLRRSDADN	45.5				
DDRRYNSHY	61.8				
DRRYNSHYY	85.9				
NGDSNANTG	48.19				

### Multi-epitope vaccine construction and processing

**Figure 5:**
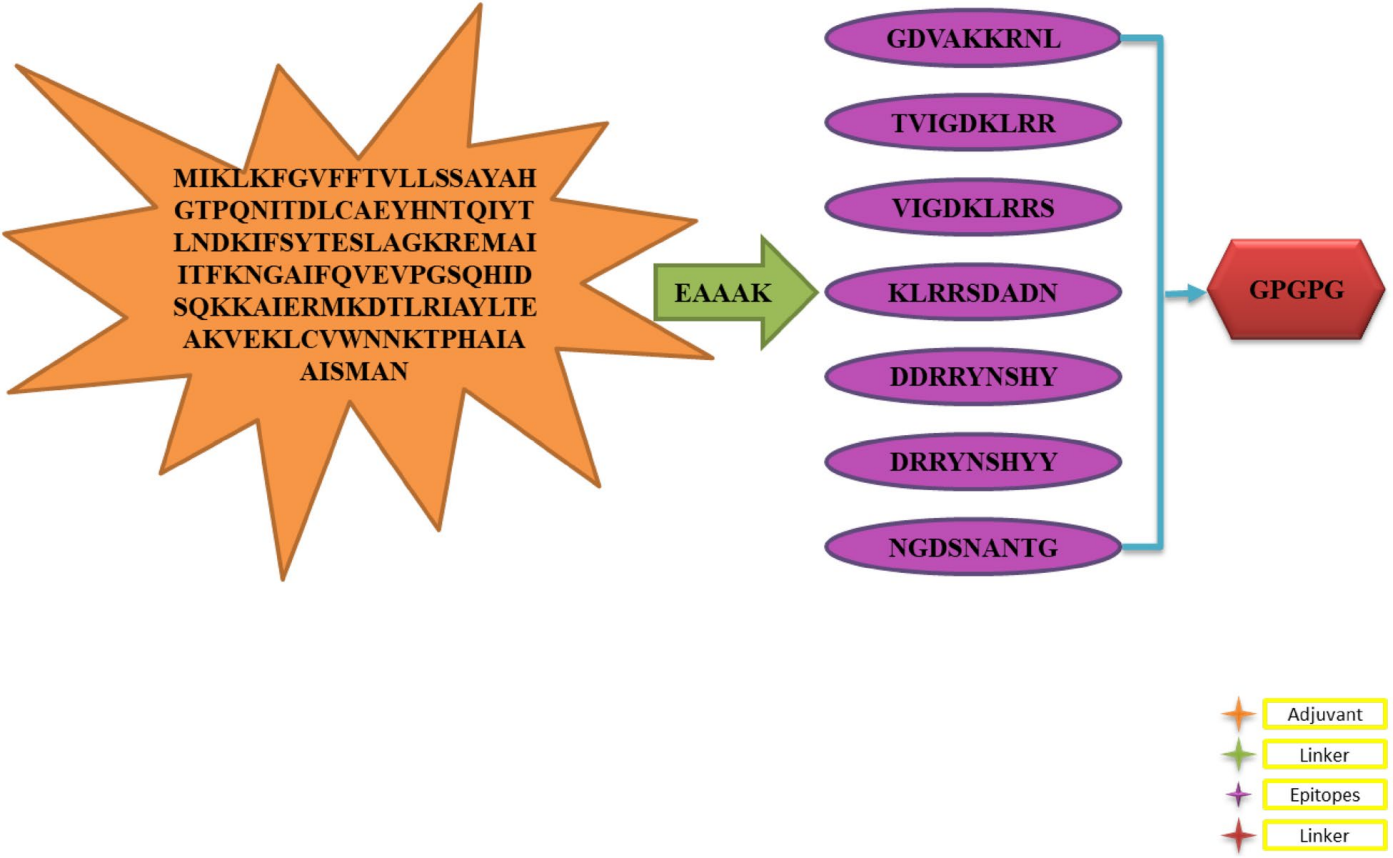
Illustration of the design of the multi-epitope vaccine construct.

An epitope-based vaccine is composed of more than one type of epitope rather than a single epitope. To overcome the limitations of single-peptide-based vaccines, which are unable to generate an effective immune response against variants of the same pathogen, the vaccine construct is designed by linking screened epitopes together through specific linkers, i.e., GPGPG linkers^55^. EAAAK is another linker that connects the adjuvant CTBS^39^ with the vaccine construct in order to increase immune efficacy^50^. In addition to their rigidity, these specific linkers enable the separation of epitopes which have been efficiently recognized by the immune system. Therefore, the designed vaccine generates an immune response that is safe, robust, and efficient. Figure 5 is schematic representation of vaccine construct.

### Structure modeling of vaccine

**Figure 6:**
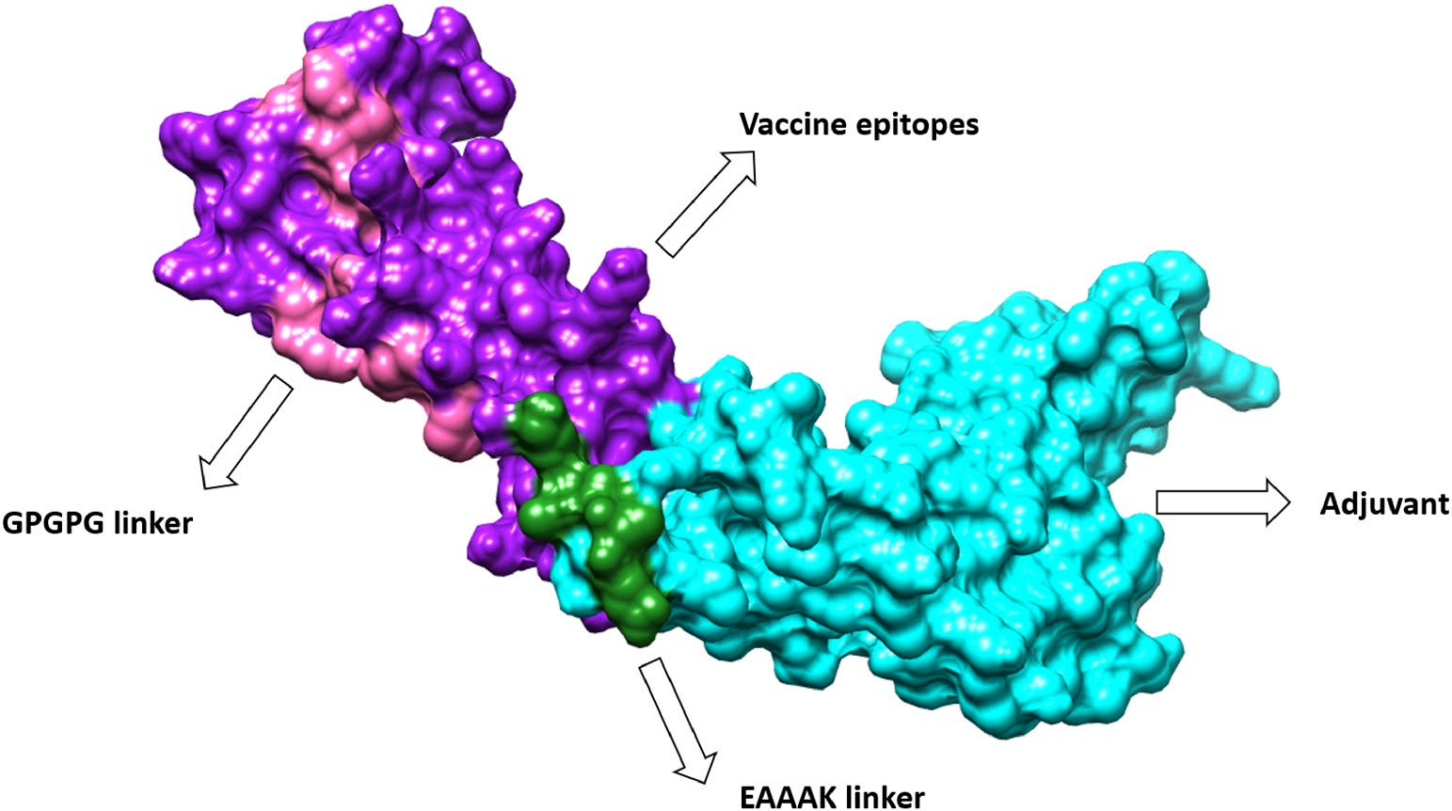
A candidate vaccine construct comprises GPGPG linkers in pink color, subunits of cholera toxin B in cyan blue color, and forest green representing EAAAK linkers, as well as vaccine epitopes are represented by purple.

3Dpro Scratch^40^ was used to model the three-dimensional structure of the vaccine. Since no appropriate template structure was available, the structure modeling was done by ab initio instead of using a homology-based or threading approach as shown in Fig. 6. Good vaccine candidates must have structural stability. During analysis, we modeled the following loops in the vaccine candidate to avoid structure instability: Cys30-Ile38, Glu50-Ile60, Ile61-Gly66, Ala67-Pro74, Thr99-Glu104, Lys105-Asn111, Aaaarg136-Gly141, Pro142-Asp148, Lys149-Gly153, Pro154-Ile159, Arg165-Leu173, Arg174-Asn180, Gly181-Gly185, Asp 186-Asn191, Gly195-Asp200, Arg201-His206, and Tyr207-Asn214.

### Disulfide engineering

**Figure 7:**
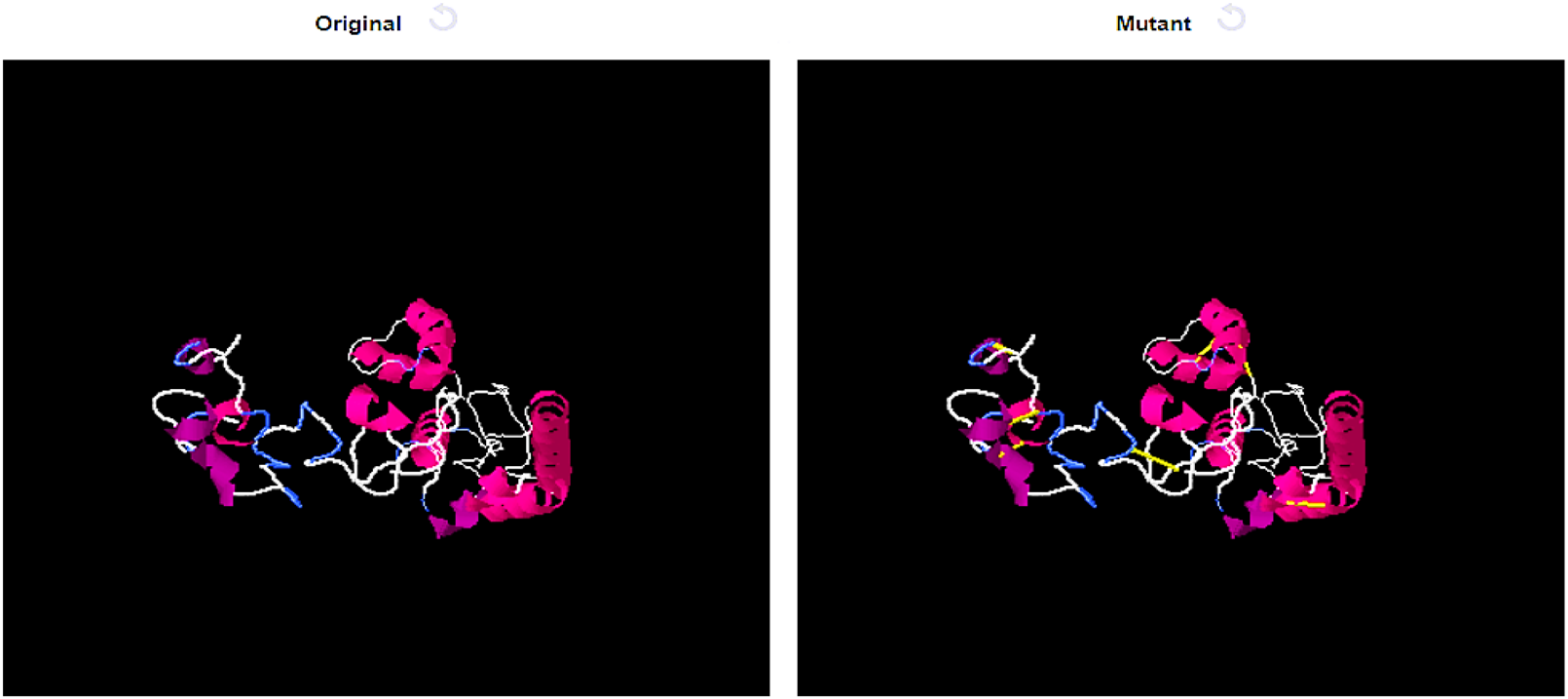
Structures of vaccines that have been mutated. Mutant structures have yellow bands which indicate the introduction of disulfide bonds.

Disulfide engineering has been used to ensure structure stability using Design 2.0^45^. Because covalent bonds keep the protein structure stable, the construct retains its geometry. Also, some amino acid residues are susceptible to degradation by enzymes. Figure 7 shows yellow sticks for those cysteine residues that are enzyme-degradable amino acids.

### Codon optimization

Using codon optimization, one can make sure that the construct's codons make use of the host immune system as efficiently as possible for maximum protein production. Maximal expression and production of proteins, CAI values between 105 and 107 have been estimated to be ideal^52^.

### Analysis of molecular docking

To activate cellular and humoral immunity, both the designed vaccine construct and the host's innate and adaptive immune cells should interact with each other. The vaccine was therefore docked with the host immune receptor in order to estimate its binding affinity. The binding affinities with receptors of the host were analyzed using a blind docking approach^56^ with MHC-I, MHC-II, and TLR-4. S-Table 1, S-Table 2, and S-Table 3 provide a summary of the results obtained from the PatchDock server, which produced 20 solutions.

### Refinement of docked complexes

PatchDock results have also been refined. UCSF Chimera 1.13.1^57^ was used to further study the binding modes and interactions of the complexes that had the lowest global energy. We chose the top docked solutions for all receptors investigated. Among the solutions for MHC-I, 4 was chosen because it is the one with the lowest global energy of − 1.76 kJ.mol^−1^, with the best contribution from ace energy (0.47 kJ. mol-1), attractive van der Waals (-5.92 kJ. mol-1), and hydrogen bond energy (-o.97 kJ. mol-1). In addition, we selected solutions 10 and 8 for MHC-II and TLR-4 on the basis of the criteria above. FireDock generated docked solutions for rescoring shown in S-Table 4, S-Table 5, and Table 6.

**Table 6:** Residues interaction of MHC-I MHC-II and TLR-4 with the final vaccine model.

Vaccine complex	Interactive residues
MHC-I	Ile7, His13, Ile35, Arg44, His197, Asp220, Gln141, Asn174, Arg256, Val49, Glu50, Trp60, Leu64, Lys75, Gln89, Trp204, Glu198, Pro210, Val248, Pro269, Arg234
MHC-II	His16, Cys30, Ala74, Leu67, Met36, Asp142, Arg44, Phe155, Gln174, Thr104, Ser88, Thr80, Ile82, Pro56, Thr77, Phe198, Gln10, Asn84, Asp171
TLR-4	Ser120, Cys390, Gly123, Leu406, Ser415, Asp428, Lys91, Kdo206, Ile91, Gln73, Ser 139, Myr205, Gln430, Asp99, His256, Val157, Leu378, Ala 137, Ile48, Val135, Cys88

### Docked confirmation of vaccine with immune receptors

**Figure 8:**
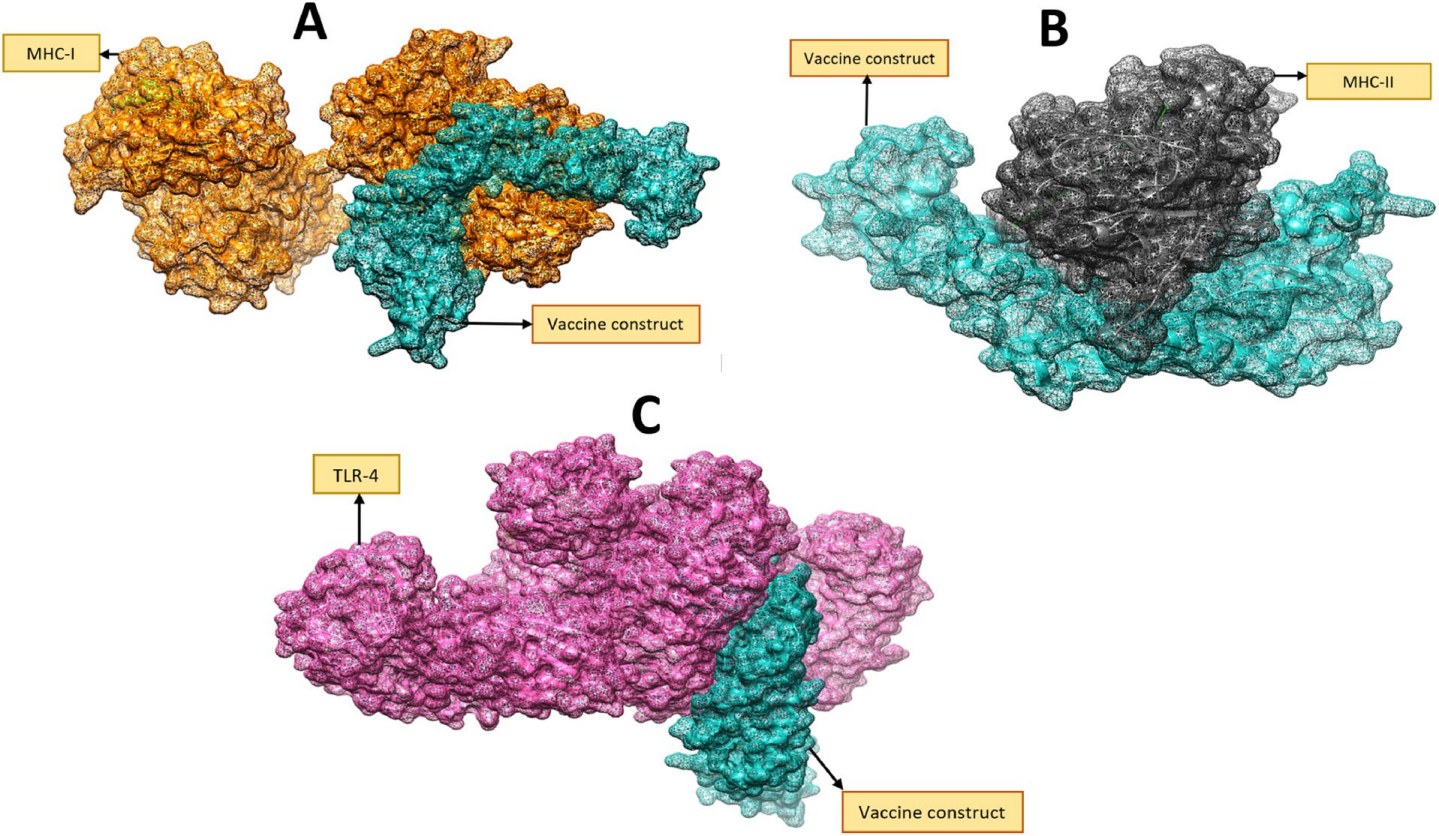
Illustration of interaction with MHC-I (**A**), MHC-II (**B**) and TLR-4 (**C**) with the vaccine.

We have visualized the best-docked complexes for each immune receptor as illustrated in Fig. 8. This tight binding exposes epitopes, enabling the immune system to recognize and process them. Immune pathways can be stimulated by vaccine epitopes, which further implies that strong and protective immune responses can be formed.

### Interactions of vaccine to immune receptors

In order to generate appropriate immune responses, vaccines need to interact properly with immune cell receptors on the host. By using Different residues of MHC-I interacted with the model vaccine construct. In molecular docking, we utilized specific residue-wise interactions between MHC-I, MHC-II, and TLR-4 receptors on vaccine constructs. Similarly, the vaccine created a significant interaction network with the MHC-II molecule^58^. In addition to hydrogen bonds and salt bridges, van der Waals interactions are also all occurring at close distances. The TLR-4 protein belongs to the TLR family of proteins that initiates adaptive and acquired immune responses. In the following Table 6 you will find the residues that interact with TLR-4, MHC I and MHC II of the model vaccine.

### Molecular dynamic simulation

**Figure 9:**
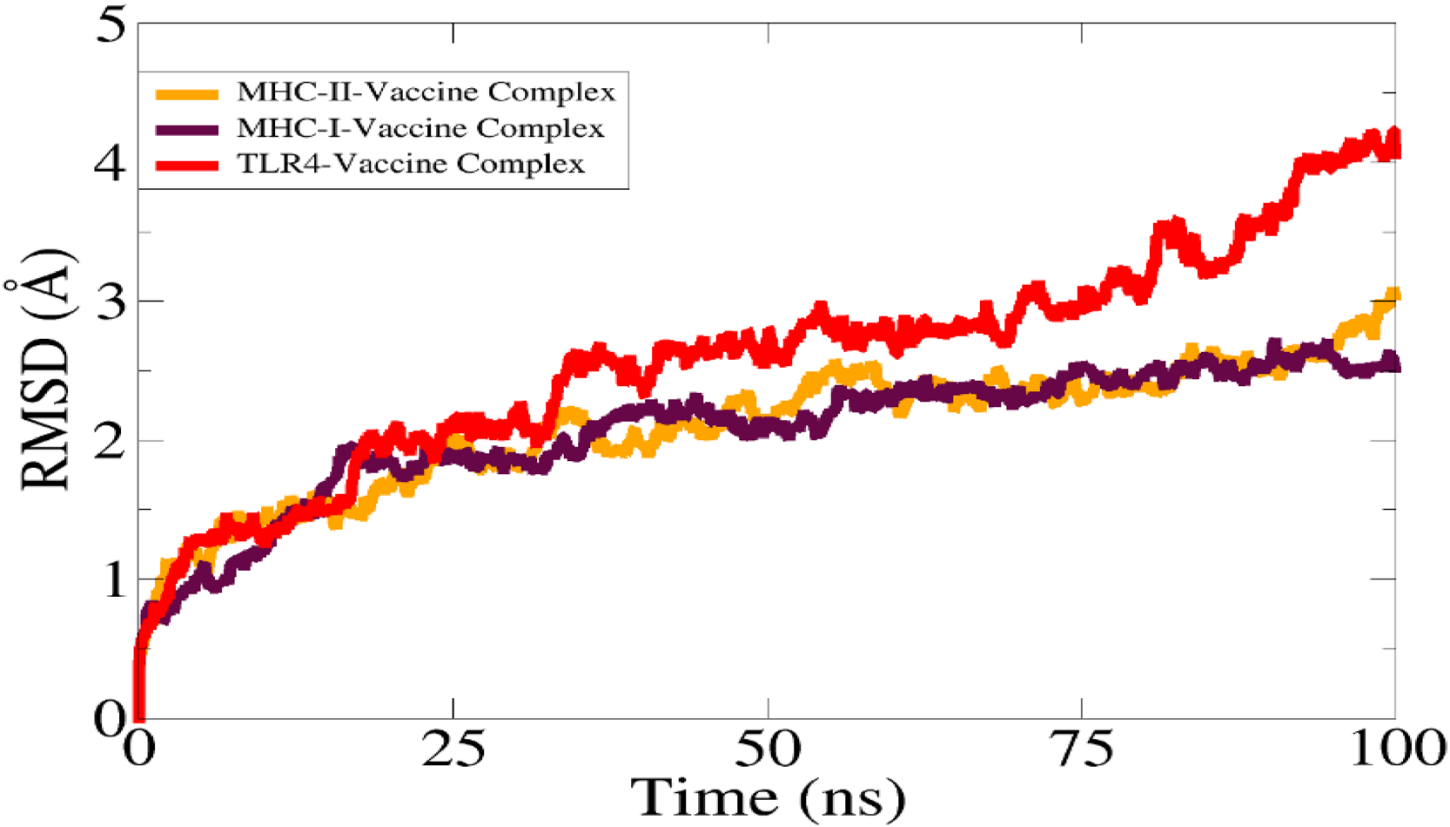
RMSD graph for analyzing simulations of trajectory.

For analyzing the dynamic behavior of macromolecules, molecular dynamics simulations are used in silico. It is allowed for atoms and molecules to interact for a specified period of time, giving an idea of how a system "evolves." There was an in-depth analysis of the docked complexes over a period of 250 ns, however, it is important to measure the binding affinity of the vaccine constructs to dock with receptors over a given period of time^59^. Nevertheless, it is imperative to make sure that the antigens in the vaccine are exposed and recognized by the immune system so it can develop an adequate immune response. The simulation period showed no drastic changes as shown in Fig. 9. RMSD in terms of carbon alpha atoms was the first analysis conducted. Each system's RMSD increased over time. When the vaccine was used with MHC-I and MHC-II, binding was lower than that with TLR-4. RMSD values for TLR4 vaccine shows deviation which is 4 Å because of high loops number and larger system size and RMSD value for MHC-I and MHC-II vaccines are 2.5 Å and 2.7 Å, respectively.

### Calculation of binding free energies

Dock complexes binding free energies were calculated using the MM-GB/PBSA approach^50^. There were − 349.45 kcal/mol free binding affinities for the TLR4 receptor and vaccine construct, as well as − 194.72 kcal/mol free binding affinities for MHC-I and the vaccine construct, and − 188.28 kcal/mol free binding affinities for MHC-II. Complex formation is most favorable in the MM-GB/PBSA when electrostatic and van der Waal energies combine. All three complexes show a dominant gas phase energy, while the polar energy is non-favorable as well. Table 7 summarizes the binding energy terms for the different complexes.

**Table 7:** Overview of the differences in binding free energies between vaccines and their receptors and are given in kcal/mol.

Energy parameter	TLR-4-vaccine complex	MHC-I-vaccine complex	MHC-II-vaccine complex
**MM-GBSA**			
VDWAALS	− 255.58	− 117.66	− 111.22
EEL	− 110.97	− 93.84	− 79.82
EGB	46.48	39.17	22.34
ESURF	− 29.38	− 22.39	− 25.10
Delta G gas	− 366.55	− 211.5	− 191.04
Delta G solv	17.1	16.78	− 2.76
Delta Total	− 349.45	− 194.72	− 188.28
**MM-PBSA**			
VDWAALS	− 255.58	− 117.66	− 111.22
EEL	− 110.97	− 93.84	− 79.82
EPB	48.11	37.15	24.28
ENPOLAR	− 30.23	− 20.22	− 21.00
Delta G gas	− 366.55	− 211.5	− 191.04
Delta G solv	17.88	16.93	3.28
Delta Total	− 348.67	− 194.57	− 187.76

## Discussion

Microbes are developing multidrug resistance quickly and are infecting people and other organisms with deadly infections. As bacteria evolve, their efficacy decreases, and AMR begins to develop as a result^60,61^. Many pathogens require significant candidate vaccines because of the occurrence of adverse effects associated with different antibiotic therapy measures. A range of infective microorganisms was employed to anticipate and discover possible new antigenic vaccine candidates through *"*in silico" subtractive proteomics. It takes a long time to conduct an experimental vaccination. Furthermore, computational methods combined with advances in genomic sciences have significantly reduced the time and resources that are required to develop vaccines against AMR pathogens. Computer analyses, in conjunction with the accessibility of complete genome sequences, suggest that we might be able to carry out an "in silico*"* subtractive proteome analysis to identify prospective vaccine candidates for *K. aerogenes*. The study also indicates several therapeutic target priority parameters^53^.

Indole negative, Gram-negative, catalase-positive, oxidase-negative, *Klebsiella aerogenes* are oxidase-negative and rod-shaped bacterium that has peritrichous flagella that allows them to move^62^. *K. aerogenes* is an opportunistic pathogen that causes nosocomial infections. Intensive care unit outbreaks are associated with it. This bacterium has originally been called *K. aerogenes*. In human feces and water, *K. aerogenes* are a strain that produces gas, is non-motile, and is often encapsulated. At present, Klebsiella pathogens cannot be treated by immunological methods, although immunological treatments have been developed successfully for other Gram-negative bacteria obtained in hospital settings. There is a 50% mortality rate, even when antimicrobial treatments are used^63^.

Most of these bacteria are susceptible to most antibiotics designed for them, but *K. aerogene* inducible resistance mechanisms are a challenge, particularly lactamases, which cause them to rapidly develop resistance to standard antibiotics during treatment. Healthy people generally do not get sick from aerogenes, which are generally found in the gastrointestinal tract^64^. As a result of the high number of nosocomial *Klebsiella* infections in developing countries. *K. aerogenes* vaccines are being developed to prevent *K. aerogenes* infection^65^.

In the past, vaccines prevented millions of cases of infectious diseases, saving lives. Successful vaccines include those used to prevent Spanish flu and smallpox, which prevented pandemic mortality^66^.

The study examined 2 vaccine targets, which includes Fe2^+−^ enterobactin ABC transporter substrate-binding protein and fimbrial biogenesis outer membrane usher protein. There were enzymes found that could be used for vaccines that met all requirements. Therefore, a wide range of pathogens can be included in the development of vaccines. It has also been established that these proteins are found on the surface of the pathogen. They are quickly recognized by the host defense system. Because of their antigenic determinants, these proteins are also immunostimulatory^67^. A further advantage is that the selected proteins do not belong to human proteomes, as similarity with human proteomes leads to potential resistance to autoimmune reactions. Additionally, antibodies are antigenic and able to bind to acquired immunity proteins and activate immune signaling pathways. Identified antigenic epitopes in the proteins have a strong binding affinity for DRB*0101 alleles, and they do not appear to be toxic or allergic. This allele is present in most human populations, causing quick and accurate responses of the immune system. To overcome the limitations of single peptide vaccines, multiepitope vaccines can be designed with predicted epitopes. The created vaccine was stable with respect to different immune receptors as well as binding to MHC-I, MHC-II, and TLR4. The enzymes identified met all the necessary requirements to be considered vaccine candidates. It ensures that the vaccine is designed to cover a variety of pathogens. It has also been established that these proteins are located on the pathogen's surface. This enables the immune system of the host to combat them.

The use of genomic information in computer-assisted vaccine design continues to gain popularity as vaccine development advances. Furthermore, it can produce results in a relatively short period of time, both saving time and money. The vaccine designed, based on the results, is a good candidate for both in vitro and in vivo testing.

## Conclusion and limitations

This study is attempting to develop a multi-epitope vaccine against *K. aerogene*, a bacterial pathogen. A variety of computer-aided approaches are being employed to develop it, including reverse vaccinology, subtractive proteomic analysis, immune-informatics, and biophysical analyses. Two potential targets were used to predict vaccine epitopes; Fe2^+−^ enterobactin ABC transporter substrate-binding protein and fimbrial biogenesis outer membrane usher protein. A variety of computer-aided approaches are being employed to develop it, including reverse vaccinology, subtractive proteomic analysis, immune-informatics, and biophysical analyses. Its antigens and epitopes also do not cause allergies and have a high affinity for binding to B-cells and T-cells. Immune responses after vaccination were simulated and revealed primary, secondary, and tertiary responses. Based on the results of all these studies, the vaccine appears to be a suitable candidate for in vivo testing for antigen-specific immunity. A vaccine against *K. aerogene* may be developed more quickly based on the findings and data of this study. The selection of our selection criteria throughout the study was quite thorough, but there are still some concerns that need to be addressed in future studies. Furthermore, the vaccine does not consider the order of the epitopes when evaluating its efficacy of vaccination. It has not been tested extensively whether MHC epitope prediction algorithms are accurate.

## Supplementary Information

Supplementary Information.

## Data Availability

The data presented in this study are available within the article.

## References

[1] Hutchings M, Truman A, Wilkinson B (2019). Antibiotics: past, present and future. Curr. Opin. Microbiol..

[2] Negahdaripour M (2018). Structural vaccinology considerations for in silico designing of a multi-epitope vaccine. Infect. Genet. Evol..

[3] MacLean, R. C. & San Millan, A. The evolution of antibiotic resistance. *Science.* **365**, 1082–1083 (2019). 10.1126/science.aax3879 31515374

[4] Covián C (2019). BCG-induced cross-protection and development of trained immunity: Implication for vaccine design. Front. Immunol..

[5] Okafor, C. N., Rewane, A. & Momodu, I. I. Bacillus Calmette Guerin. (2019). 30844212

[6] Dar HA (2019). Immunoinformatics-aided design and evaluation of a potential multi-epitope vaccine against Klebsiella pneumoniae. Vaccines.

[7] Gasperini G (2021). Salmonella paratyphi a outer membrane vesicles displaying Vi polysaccharide as a multivalent vaccine against enteric fever. Infect. Immun..

[8] ul Qamar, M. T. *et al.* Reverse vaccinology assisted designing of multiepitope-based subunit vaccine against SARS-CoV-2. *Infect. Dis. Pov.* **9**, 1–14 (2020). 10.1186/s40249-020-00752-w PMC7492789 32938504

[9] Gagneux-Brunon A, Lucht F, Launay O, Berthelot P, Botelho-Nevers E (2018). Vaccines for healthcare-associated infections: present, future, and expectations. Expert Rev. Vaccines.

[10] Bidmos, F. A., Siris, S., Gladstone, C. A. & Langford, P. R. Bacterial vaccine antigen discovery in the reverse vaccinology 2.0 era: Progress and challenges. *Front. Immunol.* **9**, (2018). 10.3389/fimmu.2018.02315 PMC6187972 30349542

[11] Saadi M, Karkhah A, Nouri HR (2017). Development of a multi-epitope peptide vaccine inducing robust T cell responses against brucellosis using immunoinformatics based approaches. Infect. Genet. Evol..

[12] Serruto D, Bottomley MJ, Ram S, Giuliani MM, Rappuoli R (2012). The new multicomponent vaccine against meningococcal serogroup B, 4CMenB: immunological, functional and structural characterization of the antigens. Vaccine.

[13] Tindall, B. J., Sutton, G. & Garrity, G. M. Enterobacter aerogenes Hormaeche and Edwards 1960 (Approved Lists 1980) and Klebsiella mobilis Bascomb et al. 1971 (Approved Lists 1980) share the same nomenclatural type (ATCC 13048) on the Approved Lists and are homotypic synonyms, with consequences for. *Int. J. Syst. Evol. Microbiol.* **67**, 502–504 (2017). 10.1099/ijsem.0.001572 27902205

[14] Davin-Regli A, Pagès J-M (2015). Enterobacter aerogenes and Enterobacter cloacae: Versatile bacterial pathogens confronting antibiotic treatment. Front. Microbiol..

[15] Jacoby, G. AmpC B-Lactamases. *Clin*. *Microbiol Rev Jan* **22**, 161–182 (2009). 10.1128/CMR.00036-08 PMC2620637 19136439

[16] Anastay M, Lagier E, Blanc V, Chardon H (2013). Épidémiologie des bêtalactamases à spectre étendu (BLSE) chez les entérobactéries dans un hôpital du sud de la France, 1999–2007. Pathol. Biol..

[17] Chang, S. C., Chen, Y. C. & Hsu, L. Y. Epidemiologic study of pathogens causing nosocomial infections. *J. Formos. Med. Assoc. Taiwan yi zhi* **89**, 1023–1030 (1990). 1982123

[18] Cantón R (2002). Epidemiology of extended-spectrum β-lactamase-producing Enterobacter isolates in a Spanish hospital during a 12-year period. J. Clin. Microbiol..

[19] Reker D, Rodrigues T, Schneider P, Schneider G (2014). Identifying the macromolecular targets of de novo-designed chemical entities through self-organizing map consensus. Proc. Natl. Acad. Sci..

[20] Pruitt KD, Tatusova T, Maglott DR (2007). NCBI reference sequences (RefSeq): a curated non-redundant sequence database of genomes, transcripts and proteins. Nucleic Acids Res..

[21] Pérez de la Lastra, J. M., Asensio-Calavia, P., González-Acosta, S., Baca-González, V. & Morales-delaNuez, A. Bioinformatic analysis of genome-predicted bat cathelicidins. *Molecules* **26**, 1811 (2021). 10.3390/molecules26061811 PMC8004601 33806967

[22] Chaudhari NM, Gupta VK, Dutta C (2016). BPGA—An ultra-fast pan-genome analysis pipeline. Sci. Rep..

[23] Bagheri, H., Dyer, R., Severin, A. & Rajan, H. Comprehensive analysis of non redundant protein database. *Res. Sq.* 1–9 (2019).

[24] Fu L, Niu B, Zhu Z, Wu S, Li W (2012). CD-HIT: Accelerated for clustering the next-generation sequencing data. Bioinformatics.

[25] Yu, N. Y. *et al.* PSORTb 3.0: Improved protein subcellular localization prediction with refined localization subcategories and predictive capabilities for all prokaryotes. *Bioinformatics* **26**, 1608–1615 (2010). 10.1093/bioinformatics/btq249 PMC2887053 20472543

[26] Masignani V, Pizza M, Moxon ER (2019). The development of a vaccine against meningococcus B using reverse vaccinology. Front. Immunol..

[27] Ahmad S, Ranaghan KE, Azam SS (2019). Combating tigecycline resistant Acinetobacter baumannii: A leap forward towards multi-epitope based vaccine discovery. Eur. J. Pharm. Sci..

[28] Chand, Y. & Singh, S. Prioritization of potential vaccine candidates and designing a multiepitope-based subunit vaccine against multidrug-resistant Salmonella Typhi str. CT18: A subtractive proteomics and immunoinformatics approach. *Microb. Pathog.* 105150 (2021). 10.1016/j.micpath.2021.105150 34425197

[29] ProtParam, E. ExPASy-ProtParam Tool.[Google Scholar]. (2017).

[30] Hossan MI (2021). Immunoinformatics aided-design of novel multi-epitope based peptide vaccine against Hendra henipavirus through proteome exploration. Inform. Med. Unlocked.

[31] Tusnady GE, Simon I (2001). The HMMTOP transmembrane topology prediction server. Bioinformatics.

[32] Sanches RCO (2021). Immunoinformatics design of multi-epitope peptide-based vaccine against Schistosoma mansoni using transmembrane proteins as a target. Front. Immunol..

[33] Doytchinova IA, Flower DR (2007). VaxiJen: a server for prediction of protective antigens, tumour antigens and subunit vaccines. BMC Bioinf..

[34] Dimitrov, I., Bangov, I., Flower, D. R. & Doytchinova, I. AllerTOP v. 2—a server for in silico prediction of allergens. *J. Mol. Model.* **20**, 2278 (2014). 10.1007/s00894-014-2278-5 24878803

[35] Adeoti OM (2021). Prediction of multi-epitopic domains of a putative oral vaccine against hepatitis C virus. Int. J. Immunol. Microbiol..

[36] Vita R (2018). The immune epitope database (IEDB): 2018 update. Nucleic Acids Res..

[37] Dhanda SK (2019). IEDB-AR: Immune epitope database—Analysis resource in 2019. Nucleic Acids Res..

[38] Aldakheel, F. M. *et al.* Proteome-wide mapping and reverse vaccinology approaches to design a multi-epitope vaccine against clostridium perfringens. *Vaccines* **9**, (2021). 10.3390/vaccines9101079 PMC8539331 34696187

[39] Stratmann T (2015). Cholera toxin subunit B as adjuvant––An accelerator in protective immunity and a break in autoimmunity. Vaccines.

[40] Ojha R, Pareek A, Pandey RK, Prusty D, Prajapati VK (2019). Strategic development of a next-generation multi-epitope vaccine to prevent Nipah Virus zoonotic infection. ACS Omega.

[41] Ismail S, Ahmad S, Azam SS (2020). Vaccinomics to design a novel single chimeric subunit vaccine for broad-spectrum immunological applications targeting nosocomial Enterobacteriaceae pathogens. Eur. J. Pharm. Sci..

[42] Giardine B (2005). Galaxy: A platform for interactive large-scale genome analysis. Genome Res..

[43] Heo L, Park H, Seok C (2013). GalaxyRefine: Protein structure refinement driven by side-chain repacking. Nucleic Acids Res..

[44] Craig, D. B. & Dombkowski, A. A. Disulfide by Design 2.0: a web-based tool for disulfide engineering in proteins. *BMC Bioinf.* **14**, 346 (2013). 10.1186/1471-2105-14-346 PMC3898251 24289175

[45] Dombkowski AA, Sultana KZ, Craig DB (2014). Protein disulfide engineering. FEBS Lett..

[46] Arumugam S, Varamballi P (2021). In-silico design of envelope based multi-epitope vaccine candidate against Kyasanur forest disease virus. Sci. Rep..

[47] Devi A, Chaitanya NSN (2021). In silico designing of multi-epitope vaccine construct against human coronavirus infections. J. Biomol. Struct. Dyn..

[48] Schneidman-Duhovny D, Inbar Y, Nussinov R, Wolfson HJ (2005). PatchDock and SymmDock: Servers for rigid and symmetric docking. Nucleic Acids Res..

[49] Mashiach E, Schneidman-Duhovny D, Andrusier N, Nussinov R, Wolfson HJ (2008). FireDock: A web server for fast interaction refinement in molecular docking. Nucleic Acids Res..

[50] Aslam, S. *et al.* Designing a multi-epitope vaccine against chlamydia trachomatis by employing integrated core proteomics, immuno-informatics and in silico approaches. *Biology (Basel).* **10**, 997 (2021). 10.3390/biology10100997 PMC8533590 34681096

[51] Lee J (2020). CHARMM-GUI supports the Amber force fields. J. Chem. Phys..

[52] Kar T (2020). A candidate multi-epitope vaccine against SARS-CoV-2. Sci. Rep..

[53] Chakkyarath V, Shanmugam A, Natarajan J (2021). Prioritization of potential drug targets and antigenic vaccine candidates against Klebsiella aerogenes using the computational subtractive proteome-driven approach. J. Prot. Proteom..

[54] Guan P, Doytchinova IA, Zygouri C, Flower DR (2003). MHCPred: A server for quantitative prediction of peptide–MHC binding. Nucleic Acids Res..

[55] Umar, A. *et al.* Development of a candidate multi-epitope subunit vaccine against klebsiella aerogenes: Subtractive proteomics and immuno-informatics approach. *Vaccines* **9**, (2021). 10.3390/vaccines9111373 PMC8624419 34835304

[56] Jalal, K. *et al.* Pan-genome reverse vaccinology approach for the design of multi-epitope vaccine construct against escherichia albertii. *Int. J. Mol. Sci.* **22**, (2021). 10.3390/ijms222312814 PMC8657462 34884620

[57] Pettersen EF (2004). UCSF Chimera—A visualization system for exploratory research and analysis. J. Comput. Chem..

[58] Ismail, S. *et al.* Pan-vaccinomics approach towards a universal vaccine candidate against WHO priority pathogens to address growing global antibiotic resistance. *Comput. Biol. Med.* 104705 (2021). 10.1016/j.compbiomed.2021.104705 34340127

[59] Nain Z (2020). Proteome-wide screening for designing a multi-epitope vaccine against emerging pathogen Elizabethkingia anophelis using immunoinformatic approaches. J. Biomol. Struct. Dyn..

[60] Roth N (2019). The application of antibiotics in broiler production and the resulting antibiotic resistance in Escherichia coli: A global overview. Poult. Sci..

[61] van Houten CB (2019). Antibiotic misuse in respiratory tract infections in children and adults—A prospective, multicentre study (TAILORED Treatment). Eur. J. Clin. Microbiol. Infect. Dis..

[62] Guo X (2018). Establishment of a molecular serotyping scheme and a multiplexed luminex-based array for Enterobacter aerogenes. Front. Microbiol..

[63] Umar A (2021). Development of a candidate multi-epitope subunit vaccine against Klebsiella aerogenes: Subtractive proteomics and immuno-informatics approach. Vaccines.

[64] Sanders Jr, W. E. & Sanders, C. C. Enterobacter spp.: pathogens poised to flourish at the turn of the century. *Clin. Microbiol. Rev.* **10**, 220–241 (1997). 10.1128/cmr.10.2.220 PMC172917 9105752

[65] Lundberg U, Senn BM, Schuler W, Meinke A, Hanner M (2013). Identification and characterization of antigens as vaccine candidates against Klebsiella pneumoniae. Hum. Vaccines Immunother..

[66] Adu-Bobie J, Capecchi B, Serruto D, Rappuoli R, Pizza M (2003). Two years into reverse vaccinology. Vaccine.

[67] Monterrubio-López, G. P. & Ribas-Aparicio, R. M. Identification of novel potential vaccine candidates against tuberculosis based on reverse vaccinology. *Biomed Res. Int.* **2015**, (2015). 10.1155/2015/483150 PMC4413515 25961021

