# Supplementary material for: Identification and construction of a multi-epitopes vaccine design against Klebsiella aerogenes: molecular modeling study
Source: Sci Rep. 2022 Aug 24;12:14402. doi: 10.1038/s41598-022-18610-0 (PMC9399595; doi:10.1038/s41598-022-18610-0)
Supplement: Supplementary file 1 — Supplementary Information. [file 41598_2022_18610_MOESM1_ESM.docx]

**S-Table 1.** Top twenty vaccines with MHC-I docked to them.

| **Solution No** | **Score** | **Area** | **ACE** | **Transformation** |
| --- | --- | --- | --- | --- |
| 1 | 20110 | 3307.10 | 383.84 | -2.80 -0.78 0.92 40.84 21.56 73.02 |
| 2 | 19360 | 2612.50 | 244.36 | 0.12 0.84 2.03 -0.02 -9.12 -41.56 |
| 3 | 19154 | 3166.20 | 446.85 | -1.46 0.81 -2.32 21.12 50.12 26.95 |
| 4 | 19096 | 3743.40 | 341.46 | 0.32 0.60 1.78 4.06 24.17 8.15 |
| 5 | 19018 | 2439.50 | 173.45 | 0.42 0.56 1.08 5.04 35.11 22.67 |
| 6 | 18452 | 2553.80 | 60.72 | -1.77 0.50 1.07 71.77 17.02 37.68 |
| 7 | 18362 | 2927.70 | -40.32 | 1.00 -0.47 1.10 -34.64 9.07 -16.88 |
| 8 | 18338 | 2285.70 | 307.16 | -2.77 -0.37 0.85 25.78 -32.01 23.18 |
| 9 | 17982 | 2517.20 | 140.31 | 1.28 -0.52 1.46 -37.74 0.91 -7.38 |
| 10 | 17552 | 2552.30 | 224.17 | 2.30 -0.74 -2.80 33.30 1.71 38.23 |
| 11 | 17542 | 3557.90 | 423.31 | 2.88 0.68 -2.77 19.07 21.31 37.90 |
| 12 | 17502 | 2448.50 | 377.48 | 1.65 -0.44 -3.13 28.40 -12.26 4.54 |
| 13 | 17492 | 2562.30 | 365.22 | 2.37 -1.47 1.77 -6.79 39.63 57.89 |
| 14 | 17420 | 2691.40 | 258.67 | -1.37 -0.25 -0.17 -11.35 -40.40 8.00 |
| 15 | 17352 | 2596.00 | 369.93 | 0.06 1.14 1.80 1.49 12.12 -41.30 |
| 16 | 17322 | 4071.00 | 144.38 | -1.89 0.01 2.55 29.87 28.47 23.98 |
| 17 | 17308 | 3110.60 | 418.60 | 0.89 0.40 1.74 -20.79 -31.64 -33.42 |
| 18 | 17294 | 3053.60 | 180.65 | 1.54 -0.76 -1.33 22.05 7.20 1.95 |
| 19 | 17216 | 2968.80 | 275.29 | -0.13 -0.05 0.80 5.09 -1.43 -24.96 |
| 20 | 17206 | 2474.50 | 420.16 | -0.86 0.70 -2.05 27.57 7.60 5.77 |

**S-Table 2.** Top twenty vaccines with MHC-II docked to them.

| **Solution No** | **Score** | **Area** | **ACE** | **Transformation** |
| --- | --- | --- | --- | --- |
| 1 | 17994 | 3730.70 | 199.85 | -0.70 -1.03 0.64 76.11 67.45 -5.34 |
| 2 | 17910 | 3069.40 | 254.81 | 0.03 0.51 -0.94 93.54 92.08 -32.38 |
| 3 | 17780 | 2859.80 | 273.95 | -0.74 0.57 3.00 92.93 72.00 -26.54 |
| 4 | 17698 | 2952.80 | 337.26 | -0.59 -1.24 0.83 78.22 64.29 0.26 |
| 5 | 17620 | 3448.20 | 87.25 | 1.65 1.11 -2.88 116.43 72.98 13.52 |
| 6 | 17536 | 2821.20 | 280.09 | -2.29 -0.32 3.14 129.86 81.54 41.70 |
| 7 | 17092 | 2068.70 | 190.66 | -2.57 0.26 1.85 147.17 14.53 21.56 |
| 8 | 16984 | 3051.50 | -5.73 | -1.59 0.47 1.21 141.33 47.63 1.36 |
| 9 | 16938 | 2820.80 | 165.23 | 1.02 -0.70 0.24 75.80 88.20 -9.08 |
| 10 | 16938 | 2730.50 | 166.33 | 0.17 1.07 -1.23 134.94 75.04 -7.32 |
| 11 | 16916 | 3200.60 | 313.01 | 1.32 -0.00 1.80 76.97 60.45 -7.37 |
| 12 | 16676 | 2576.00 | 334.54 | 0.37 0.50 -1.06 101.37 99.66 -33.32 |
| 13 | 16646 | 2086.20 | 217.94 | 2.72 -0.55 -2.36 89.82 81.34 39.69 |
| 14 | 16642 | 2531.20 | 396.80 | -0.74 -0.93 0.06 96.50 26.21 -21.43 |
| 15 | 16634 | 2631.20 | 406.80 | -0.87 0.42 -0.35 74.35 69.94 -17.85 |
| 16 | 16624 | 2590.20 | 192.81 | 2.51 -0.18 -2.21 102.95 88.07 40.09 |
| 17 | 16564 | 2123.10 | 236.68 | 1.02 0.34 -2.30 162.28 70.05 4.20 |
| 18 | 16512 | 2858.90 | -103.93 | 2.19 -0.34 2.95 121.38 28.32 17.21 |
| 19 | 16422 | 2363.30 | 263.19 | 2.68 -0.72 -2.18 87.17 76.36 36.44 |
| 20 | 16348 | 2629.50 | 439.74 | 1.42 -0.02 1.27 80.39 62.27 -20.07 |

**S-Table 3.** Top twenty vaccines with TLR-4 docked to them.

| **Solution No** | **Score** | **Area** | **ACE** | **Transformation** |
| --- | --- | --- | --- | --- |
| 1 | 24054 | 3749.50 | 457.26 | 0.19 -1.04 2.51 -0.04 -3.93 -47.41 |
| 2 | 22210 | 3332.00 | 59.05 | -0.91 0.54 1.01 25.23 12.79 -37.47 |
| 3 | 21980 | 3759.20 | -2.23 | 1.40 -0.89 -1.99 4.18 5.19 16.84 |
| 4 | 21902 | 3241.40 | 326.15 | 1.79 0.88 -0.94 -7.11 18.31 -63.74 |
| 5 | 21674 | 3129.20 | 256.64 | -1.99 0.65 -3.04 4.28 37.26 -70.12 |
| 6 | 20104 | 3771.00 | -189.57 | -1.88 0.52 -2.86 -0.99 36.55 -70.20 |
| 7 | 19774 | 2722.10 | 108.25 | 1.28 0.40 1.25 -52.93 -1.72 -0.99 |
| 8 | 19772 | 2921.10 | 499.00 | -1.90 -0.60 2.75 24.48 45.92 -44.84 |
| 9 | 19756 | 3128.20 | 459.72 | -2.83 0.66 3.00 13.01 -14.15 15.57 |
| 10 | 19692 | 3635.60 | 140.65 | 0.39 -1.06 2.49 -3.77 -6.84 -47.16 |
| 11 | 19486 | 2855.60 | 271.20 | 1.31 0.20 -0.79 -13.87 16.44 -62.54 |
| 12 | 19184 | 3226.30 | 50.24 | -2.75 -0.15 -0.98 -41.44 -6.85 55.09 |
| 13 | 18956 | 3713.30 | 492.01 | 1.20 0.98 1.44 -54.30 1.55 -14.53 |
| 14 | 18634 | 2774.40 | 353.16 | -0.21 0.83 0.87 4.65 48.49 -41.07 |
| 15 | 18620 | 3354.00 | 481.44 | 1.61 1.30 2.48 -55.78 -11.99 -54.17 |
| 16 | 18580 | 2688.00 | 488.57 | -0.28 -1.00 0.41 -39.70 -40.92 -28.25 |
| 17 | 18454 | 2986.00 | 107.69 | -1.47 0.63 0.73 0.39 -14.37 -22.63 |
| 18 | 18242 | 2371.40 | 191.32 | -2.25 -0.17 2.20 -20.68 3.94 3.97 |
| 19 | 18162 | 2832.70 | 347.30 | -1.92 -0.76 1.86 -15.03 4.02 4.15 |
| 20 | 18020 | 3244.00 | 39.05 | 1.71 -0.32 0.52 -30.43 31.94 -35.99 |

**S-Table 4.** FireDock rescoring solutions of MHC-I-vaccine expressed in terms of energy in KJ.m-1.

| **Rank** | [**Solution Number**](http://bioinfo3d.cs.tau.ac.il/FireDock/bin/showRes.pl?id=1i1yMHCI.pdb_Refinedvaccinemodel5.pdb_15_43_8_30_3_122&from=1&to=20&sortBy=1) | [**Global Energy**](http://bioinfo3d.cs.tau.ac.il/FireDock/bin/showRes.pl?id=1i1yMHCI.pdb_Refinedvaccinemodel5.pdb_15_43_8_30_3_122&from=1&to=20&sortBy=2) | [**Attractive VdW**](http://bioinfo3d.cs.tau.ac.il/FireDock/bin/showRes.pl?id=1i1yMHCI.pdb_Refinedvaccinemodel5.pdb_15_43_8_30_3_122&from=1&to=20&sortBy=3) | [**Repulsive VdW**](http://bioinfo3d.cs.tau.ac.il/FireDock/bin/showRes.pl?id=1i1yMHCI.pdb_Refinedvaccinemodel5.pdb_15_43_8_30_3_122&from=1&to=20&sortBy=4) | [**ACE**](http://bioinfo3d.cs.tau.ac.il/FireDock/bin/showRes.pl?id=1i1yMHCI.pdb_Refinedvaccinemodel5.pdb_15_43_8_30_3_122&from=1&to=20&sortBy=5) | [**HB**](http://bioinfo3d.cs.tau.ac.il/FireDock/bin/showRes.pl?id=1i1yMHCI.pdb_Refinedvaccinemodel5.pdb_15_43_8_30_3_122&from=1&to=20&sortBy=6) |
| --- | --- | --- | --- | --- | --- | --- |
| 1 | 4 | -1.76 | -5.92 | 2.31 | 0.47 | -0.97 |
| 2 | 3 | 4.55 | -4.14 | 0.00 | 1.89 | -0.59 |
| 3 | 1 | 6.04 | -3.34 | 0.00 | 3.04 | -0.60 |
| 4 | 8 | 9.01 | -49.30 | 70.18 | 15.53 | -3.69 |
| 5 | 6 | 13.96 | -9.80 | 0.32 | 2.33 | 0.00 |
| 6 | 9 | 19.13 | -32.63 | 23.85 | 10.75 | -4.01 |
| 7 | 10 | 31.93 | -31.68 | 19.78 | 19.58 | -6.15 |
| 8 | 5 | 35.15 | -35.51 | 91.69 | 9.23 | -4.10 |
| 9 | 2 | 433.44 | -53.73 | 582.83 | 15.71 | -5.87 |
| 10 | 7 | 1926.51 | -73.28 | 2570.71 | -5.29 | -9.49 |

**S-Table 5.** FireDock rescoring solutions of MHC-II-vaccine expressed in terms of energy in KJ.m-1.

| **Rank** | [**Solution Number**](http://bioinfo3d.cs.tau.ac.il/FireDock/bin/showRes.pl?id=1kg0MHCII.pdb_Refinedvaccinemodel5.pdb_10_43_8_30_3_122&from=1&to=20&sortBy=1) | [**Global Energy**](http://bioinfo3d.cs.tau.ac.il/FireDock/bin/showRes.pl?id=1kg0MHCII.pdb_Refinedvaccinemodel5.pdb_10_43_8_30_3_122&from=1&to=20&sortBy=2) | [**Attractive VdW**](http://bioinfo3d.cs.tau.ac.il/FireDock/bin/showRes.pl?id=1kg0MHCII.pdb_Refinedvaccinemodel5.pdb_10_43_8_30_3_122&from=1&to=20&sortBy=3) | [**Repulsive VdW**](http://bioinfo3d.cs.tau.ac.il/FireDock/bin/showRes.pl?id=1kg0MHCII.pdb_Refinedvaccinemodel5.pdb_10_43_8_30_3_122&from=1&to=20&sortBy=4) | [**ACE**](http://bioinfo3d.cs.tau.ac.il/FireDock/bin/showRes.pl?id=1kg0MHCII.pdb_Refinedvaccinemodel5.pdb_10_43_8_30_3_122&from=1&to=20&sortBy=5) | [**HB**](http://bioinfo3d.cs.tau.ac.il/FireDock/bin/showRes.pl?id=1kg0MHCII.pdb_Refinedvaccinemodel5.pdb_10_43_8_30_3_122&from=1&to=20&sortBy=6) |
| --- | --- | --- | --- | --- | --- | --- |
| 1 | 10 | -7.24 | -10.03 | 1.63 | -0.73 | -0.61 |
| 2 | 6 | -6.39 | -8.34 | 4.24 | 0.37 | -1.53 |
| 3 | 2 | -1.18 | -2.77 | 0.93 | -2.03 | 0.00 |
| 4 | 7 | 8.30 | -10.89 | 7.39 | 0.56 | -1.07 |
| 5 | 5 | 15.87 | -2.78 | 0.91 | 2.19 | 0.00 |
| 6 | 9 | 22.66 | -4.78 | 1.09 | 1.71 | 0.00 |
| 7 | 4 | 1758.37 | -37.58 | 2245.76 | 9.71 | -3.01 |
| 8 | 3 | 2754.48 | -32.31 | 3431.60 | 12.74 | -4.94 |
| 9 | 1 | 3465.00 | -57.47 | 4420.48 | -3.22 | -4.75 |
| 10 | 8 | 4274.98 | -45.36 | 5376.38 | 11.50 | -4.24 |

**S-Table 6.** FireDock rescoring solutions of TLR-4-vaccine expressed in terms of energy in KJ.m-1.

| **Rank** | [**Solution Number**](http://bioinfo3d.cs.tau.ac.il/FireDock/bin/showRes.pl?id=4g8aTLR4.pdb_Refinedvaccinemodel5.pdb_14_47_11_30_3_122&from=1&to=20&sortBy=1) | [**Global Energy**](http://bioinfo3d.cs.tau.ac.il/FireDock/bin/showRes.pl?id=4g8aTLR4.pdb_Refinedvaccinemodel5.pdb_14_47_11_30_3_122&from=1&to=20&sortBy=2) | [**Attractive VdW**](http://bioinfo3d.cs.tau.ac.il/FireDock/bin/showRes.pl?id=4g8aTLR4.pdb_Refinedvaccinemodel5.pdb_14_47_11_30_3_122&from=1&to=20&sortBy=3) | [**Repulsive VdW**](http://bioinfo3d.cs.tau.ac.il/FireDock/bin/showRes.pl?id=4g8aTLR4.pdb_Refinedvaccinemodel5.pdb_14_47_11_30_3_122&from=1&to=20&sortBy=4) | [**ACE**](http://bioinfo3d.cs.tau.ac.il/FireDock/bin/showRes.pl?id=4g8aTLR4.pdb_Refinedvaccinemodel5.pdb_14_47_11_30_3_122&from=1&to=20&sortBy=5) | [**HB**](http://bioinfo3d.cs.tau.ac.il/FireDock/bin/showRes.pl?id=4g8aTLR4.pdb_Refinedvaccinemodel5.pdb_14_47_11_30_3_122&from=1&to=20&sortBy=6) |
| --- | --- | --- | --- | --- | --- | --- |
| 1 | 8 | 1.45 | -1.06 | 0.76 | 0.31 | 0.00 |
| 2 | 2 | 6.41 | -24.70 | 26.24 | 3.64 | -3.17 |
| 3 | 1 | 9.36 | -1.75 | 1.19 | 3.85 | -0.62 |
| 4 | 5 | 138.73 | -50.46 | 157.72 | 17.04 | -5.57 |
| 5 | 9 | 556.12 | -6.95 | 673.35 | 6.97 | -1.36 |
| 6 | 7 | 758.44 | -64.42 | 1106.12 | -4.41 | -5.07 |
| 7 | 6 | 797.74 | -70.47 | 1052.70 | 0.39 | -4.58 |
| 8 | 4 | 916.11 | -78.05 | 1271.29 | 10.44 | -6.22 |
| 9 | 3 | 2010.89 | -83.90 | 2629.73 | 14.12 | -10.32 |
| 10 | 10 | 2252.48 | -89.50 | 2933.18 | 14.78 | -7.74 |
